# Los Angeles, California

**Published:** 1873-05

**Authors:** 


**Los Angeles, California.**

Thanks are due to our friend, J. H. Leal, M.D., well known to the alumni of Rush, for Los Angeles papers detailing the favorable character of that region as a sanitary resort, particularly for patients disposed to tuberculosis. Dr. Leal has himself found there the boon of good health which other climates had denied him. Our own opinion is especially favorable to this locality, in very many cases of tuberculosis, even when unmistakably developed.

We take the liberty, that we hope our friend will forgive, to refer those seeking information on this vital point to his address, Temple Block, Los Angeles, California.

